# A Novel Profiled Multi-Pin Electrospinning System for Nanofiber Production and Encapsulation of Nanoparticles into Nanofibers

**DOI:** 10.1038/s41598-020-60752-6

**Published:** 2020-03-09

**Authors:** G. T. V. Prabu, Bhaarathi Dhurai

**Affiliations:** 1 ICAR-Central Institute for Research on cotton Technology, Mumbai, India; 2 Department of Fashion Technology, Kumaraguru College of Technology, Coimbatore, India

**Keywords:** Biomaterials, Techniques and instrumentation

## Abstract

Electrospinning with various machine configurations is being used to produce polymer nanofibers with different rates of output. The use of polymers with high viscosity and the encapsulation of nanoparticles for achieving functionalities are some of the limitations of the existing methods. A profiled multi-pin electrospinning (PMES) setup is demonstrated in this work that overcomes the limitations in the needle and needleless electrospinning like needle clogging, particle settling, and uncontrolled/uneven Taylor cone formation, the requirement of very high voltage and uncontrolled distribution of nanoparticles in nanofibers. The key feature of the current setup is the use of profiled pin arrangement that aids in the formation of spherical shape polymer droplet and hence ensures uniform Taylor cone formation throughout the fiber production process. With a 10 wt% of Polyvinyl Alcohol (PVA) polymer solution and at an applied voltage of 30 kV, the production rate was observed as 1.690 g/h and average fiber diameter obtained was 160.5 ± 48.9 nm for PVA and 124.9 ± 49.8 nm for Cellulose acetate (CA) respectively. Moreover, the setup also provides the added advantage of using high viscosity polymer solutions in electrospinning. This approach is expected to increase the range of multifunctional electrospun nanofiber applications.

## Introduction

The production of electrospun nanofibers gained the attention of scientists and researchers throughout the globe recently^1^. Electrospinning as a process is well known to produce nanofibers with average fiber diameter in the range of 50 to 1000 nm spun from natural and synthetic polymers, polymers loaded with nanoparticles or active ingredients, metals, ceramics and so on^1–5^. The electrospun fibers have a high surface area to volume ratio, surface functionalities and superior mechanical properties when the diameter of the polymer fiber is reduced to submicron and nano scale^6^. The electrospun fibers find applications in different fields including air and water filtration^7–10^, tissue engineering^11,12^, sensors^13–16^, drug delivery^11,12,17–20^, wound dressing^21–23^, technical textiles^16^ and energy applications^24–26^.

In the present scenario, the development of a single matrix with multifunctional characteristics is demanded in every field of application. The incorporation of functional nanoparticles in electrospun fiber can meet the requirements of simultaneous improved mechanical, electrochemical and other technical performances^27–29^. The relationship among the material’s structure and its functional performance, and the ability to tune them are of crucial significance in designing an effective product^28^. As incorporation of nanoparticles into the polymer affects the solution properties like polymer viscosity, surface tension and solvent volatility and hence, poses difficulty in handling with the existing needle electrospinning systems to produce nanofibers, continually. In the case of needle electrospinning, sphere shape droplet is formed on the tip of the nozzle with semi-vertical angle (α), which helps to create a Taylor cone with a minimum voltage^17,18^. In spite of the advantages of needle electrospinning, such as being inexpensive and versatile, the use of it in actual applications have been restricted, due to low production rate and the problem of needle blocking, particularly when using high viscosity polymer and functional particles in the spinning solution^30^. To overcome these bottlenecks, the needleless electrospinning was invented^31^. Among several methods for mass production of nanofibers, creation of multiple jets using rotating disks^32^, conical wire coil^33^, rollers^34^, balls^14^, bubbles^35^, rod^36^, Twisted wire spinnerets^37^ and cones^38,39^ have turned the electrospinning technique, the most effective process for nanofiber production. In most of the needleless upward electrospinning, numerous jets are formed simultaneously from the liquid surfaces without the influence of capillary effect, which typically requires a high voltage upto 90 kV to form a Taylor cone and for fiber formation^40–42^. A sphere shape profile creation on the electrospinning spinneret can reduce the applied voltage and produce fine fibers with minimum voltage^40^.

In view of this, a profiled multi-pin high production electrospinning proto design is demonstrated. The developed system has the combined advantages of both needle and needleless electrospinning process. In this system, 21number of half-sphere shaped profiled multi-pins with a diameter ranging from 2 mm to 3.5 mm was used. The nanoparticles were mixed with polymer solution and loaded simultaneously on the profiled pin surfaces at periodic intervals. It creates a sphere-shaped polymer profile on the pin surface. It was found that a voltage of around 25–30 kV was sufficient enough to produce Taylor cones at high throughput rate and the resultant fibers had similar morphology as that of conventional electrospun fibers. This method can produce nanofibers using high viscosity polymer solutions without causing needle blockage. The profiled pin surface is designed to hold the nano/microparticles in the polymer solutions.

## Results and Discussion

### Issues existing with conventional electrospinning procedures

The two commonly accepted configurations of electrospinning fiber production systems are needle and needleless electrospinning. Both configurations have their advantage and disadvantages in different aspects and perspectives. The Taylor cone formation is the most important phenomenon to produce finer and even fiber diameter using the minimum voltage^40,41^. The sphere shaped polymer droplet creation and the uniform Taylor cone formation are the key advantages in needle electrospinning, that facilitates to produce smooth and finer fibers. For higher production, needleless electrospinning is an established technique in which multiple Taylor cone formation happens on the open liquid surface, and there is a continuous production of nanofibers. In recent years, a lot of research has focussed on various kinds of needleless electrospinning spinnerets. More recently, an annular spinneret electrospinning has been reported to be developed for high production of nanofibers, but limiting the applied voltage as low as 60–70 kV to produce fibers^43^. Holopainen *et al*.^37^ developed a twisted wire spinneret for high production of nanofibers. However frequent drying of polymer solution on the twisted wire was one of limitations of the system. Liu *et al*.^44^ designed a circular rotary bead wire as the needleless spinneret which could create multiple jets in the process of electrospinning. Airflow was used to improve the ring needleless electrospinning spinneret, and they have reported the effects of relative airflow on the nanofiber quality and productivity^45^. Wang *et al*.^46,47^ developed a spiral coil and multiple rings spinnerets to produce a large quantity of nanofibers. A comparative study of the disk and cylinder nozzles for needleless electrospinning shows that the disk nozzle needed a relatively low applied voltage to initiate fiber formation^32^. Jirsak *et al*.^48^ prepared polymeric acid nanofibers using roller electrostatic spinneret. Besides, a stepped pyramid-shaped spinneret had been used to prepare high production nanofibers and core-shell nanofibers^49,50^. Impact of fiber generator geometry on electric intensity, fiber diameter and productivity were examined by H. Niu, *et al*.^14,51^. But, a majority of the developed needleless electrospinning system adopts the solution bath open to the atmosphere during electrospinning. Consequently, the solvent gets evaporated and viscosity changes over the time^37,43^. When high voltage is applied to a flat liquid surface, some energy from the DC voltage will be lost in an effort to create the Taylor cone^40^. Moreover, the polymer viscosity is an essential parameter for electrospinning because the polymer droplets should be exposed into a high voltage electric field to create Taylor cone and produce fiber. Due to the flat surface of a solution with high concentration, splitting of polymer into small droplet is also a difficult task in needleless electrospinning process. Hence around 40–90 kV DC voltage is applied to various designs of needleless electrospinning spinneret to create Taylor cone and produce nanofibers (Slit surface, coil and rotating drum electrospinning etc.)^42,52^. It results in uneven Taylor cone formation. Also, very high voltage affects the uniformity and morphology of the produced fiber, and there is no control on nanoparticles while embedding them into the nanofibers during the nanofiber production process. Further, particle sedimentation can happen during the continuous running of the machine when the huge volume of nanoparticles are being mixed with polymer solution without resorting any periodic mixing^40^. So the present need is a method for production of nanoparticle encapsulated composite electrospun mat that has the even distribution of nanoparticles with uniform fiber morphology. In order to address these issues, the foremost requirement is that of a uniform sphere-shaped polymer droplet creation. Besides, the Taylor cone must be maintained as like a needle electrospinning system with improved production rates and without any blockage of the needles. Further, to produce nanoparticle encapsulated electrospun nanofibers, there should be provisions to hold/feed the nanoparticles during the fiber production, and periodic turbulence within the polymer solution to avoid settling of particles. The enclosed polymer reservoir helps to avoid atmospheric interaction with a polymer solution and hence, maintains the viscosity of the solution throughout the process.

### Nanofiber production through profiled multi-pin electrospinning method

**Figure 1:**
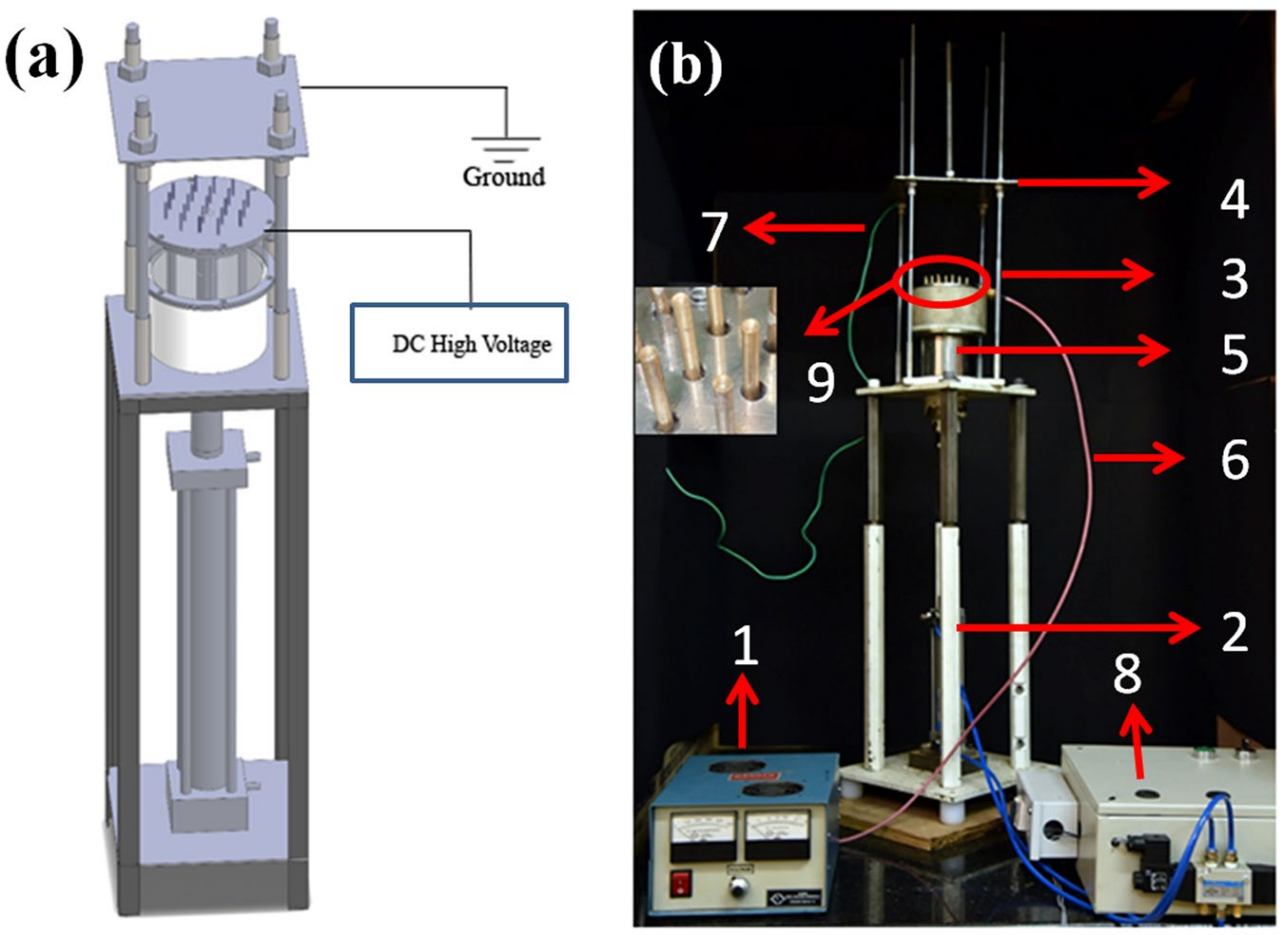
(**a**) Schematic diagram of PMES (**b**). Fabricated PMES setup; (1) high voltage power source (2) spinneret drive arrangement (3) profiled multi-pin spinneret (4) stationary flat collector. (5) polymer reservoir (6) DC high voltage cable (7) grounding cable (8) pneumatic circuit (9) profiled multi-pin magnified view.

**Figure 2:**
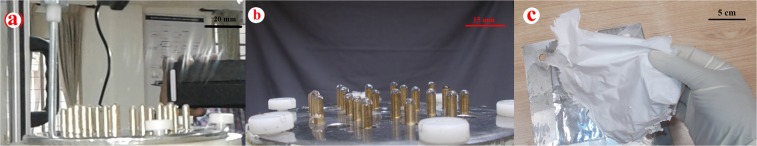
(**a**) Photograph of nanofiber emerged from the profiled multi-pin spinneret. (**b**) Sphere shaped polymer profile created on the profiled pin surface (**c**) PVA electrospun nanofiber mat produced via PMES setup.

In this method, we attempted to integrate both the needle and needleless electrospinning effect on a single profiled multi-pin electrospinning setup, to produce electrospun fibers with / without encapsulation of nanoparticles. The prototype of the profiled multi-pin spinneret electrospinning schematic diagram and fabricated setup is shown in Fig. 1a,b. The spinneret consists of 21numbers of half-sphere shaped profiled pins integrated into a circular disc with a spacing of approx 15 mm each. The polymer solution, stored in the bottom reservoir, was loaded onto the profiled pin surfaces by an up and down motion arrangement of the spinneret assembly at a prefixed time interval, known as polymer loading cycle time. As the pin surface goes through the polymer reservoir, the polymer solution experiences various forces due to gravity, viscosity and surface tension^53^. These forces determine the amount of polymer that gets engaged on the pin profile surface. In the absence of an external field, the profiled pin surface creates the sphere-shaped polymer droplet, similar to the needle electrospinning as shown in Fig. 2b. Once the splitting of polymer solution and droplet occurs, a minimum voltage is enough to initiate Taylor cone formation as compared to the flat solution surface of the needleless electrospinning, which requires very high voltage. The PMES created polymer droplet profile is similar to that created by the needle electrospinning. Hence, a minimum voltage of aound 30 kV was used for the multi-pin electrospinning to produce nanofibers (Fig. 2a nanofibers ejecting from the spinneret). Many multi-jet spinnerets have been developed to increase the production of nanofiber, but the scale up of the process still is at an early stage. Each spinneret design has its advantages and disadvantages- wires, discs and cylinders spinnerets generally increase productivity, but require very high voltage and form uneven Taylor cone resulting in production of uneven fibers. In the case of free surface electrospinning, various spinning parameters are poorly controlled^54^. The PMES method developed provides solutions to those issues and could be very much suitable to produce nanoparticle encapsulated electrospun mat to enhance the functional applications of nanofibers. The half-sphere shaped profiled multi-pins help to maintain the polymer quantity [12–15 µl] and the sphere size of the droplet during the process and make it possible to hold the particles, when mixed with a polymer solution. Hence, the sphere-shaped polymer profile formation remains undisturbed throughout the fiber production process. The size of the nanoparticles and polymer concentration does not affect the electrospinning performance of the PMES system.

### Effect of PMES spinneret behaviour on different polymeric concentrations

**Figure 3:**
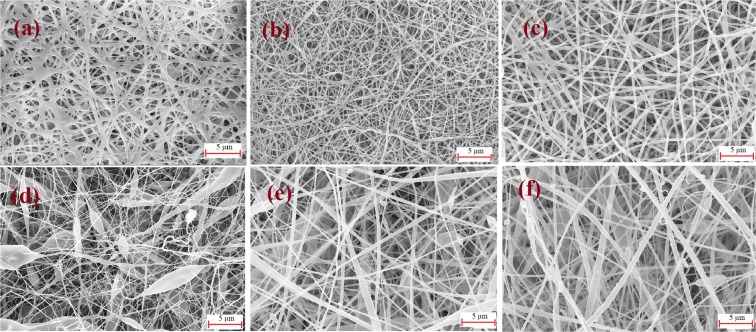
FESEM image of nanofiber produced with the profiled multi-pin electrospinning setup. The images are from PVA and CA polymers and solvents ((**a**) PVA 8 wt %, (**b**) PVA 10 wt %, (**c**) PVA 12 wt %,(**d**) CA 12 wt %, (**e**) CA 15 wt %, (**f**) CA 18 wt %).

Polymer concentration and viscosity determine the fiber diameter and morphology. Hence, two different polymer, Polyvinyl Alcohol (PVA) and Cellulose Acetate (CA), with varying concentrations were used to investigate the behaviour of the profiled multi-pin electrospinning machine. The selected concentrations were 8, 10, 12 wt % and 12, 15, 18 wt % for polyvinyl alcohol (PVA) and cellulose acetate (CA), respectively. As observed in field-emission scanning electron microscope (FESEM) images shown in Fig. 3, it is clear that at a lower polymeric concentration (CA 12 wt %), the charged jet get fragmented into discrete beads before reaching collector, due to the effect of the low entanglement of the polymeric solution, surface tension and applied voltage^55,56^. As a result, fibers with beads and uneven fiber distribution were noticed in CA 12 wt% with 30 kV connected voltage (Fig. 3d).Due to low surface tension of polymer liquid, the sphere shaped polymeric profile formation on the profiled pin surface was disturbed. Instead, a flat polymer surface was noticed and hence, necessitated slightly higher voltage about (32 kV) to generate the Taylor cone and fibers continuously^57^. Wet fiber formation was observed in PVA with 8 wt% (Fig. 3a) at 30 kV voltage is most likely due to aqueous solvent medium of PVA with a low polymeric concentration (8 wt %), the polymer was very dilute even to enable the molecules to be entangled for forming a fiber. Subsequently, the PVA solution allowed a moderate evaporation of water, and thus, enough drying was not possible with the collector distance of 12 cm. The results obtained here are similar to previously reported studies^58,59^.

At an increased polymeric concentration, the polymer chain entanglement also increased resulting in sphere shaped Taylor cone formation and hence production of finer, and even nanofibers^60,61^. The same trend was noticed for PVA 10 wt % and CA 15 wt % (Fig. 3b,e) and they were fixed at optimum polymer concentration for PVA and CA, respectively. As the polymer concentrations were increased, nanofibers were produced with an increased diameter in both the polymer solutions at 12 wt%, 18 wt % for PVA and CA, correspondingly (Fig. 3c,f). The evaluation of the PMES was carried with different levels of viscosities (300–4500 cp) of the polymer with and without zinc oxide nanoparticles. For viscosity above 3000 cP, drying of the thin solution film on the profiled pin was observed during the continuous running experiments, which has been removed by cleaning to avoid the unstable spinning process.

### Effect of experimental parameters on PMES

**Figure 4:**
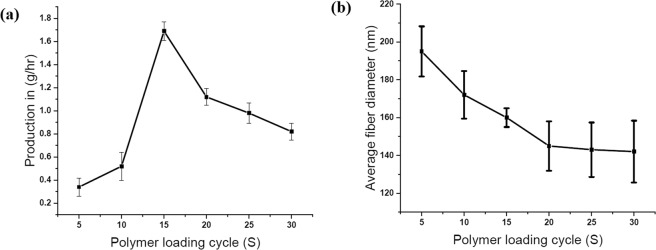
(**a**) Plot of nanofiber production based on polymer loading cycle time (**b**) plot of nanofiber diameter based on polymer loading cycle time.

The electrospinning process and machine parameters have to be controlled in order to achieve the required fiber characteristics. The profiled pin diameter, applied voltage, polymer loading cycle time, and spinneret to collector distance are the crucial parameters. Based on the preliminary trial with different diameters of the profiled pins, the diameter of the profiled pin was fixed as 3.5 mm that is capable of holding 12–15 µl spherical polymer solutions. Below the pin diameter of 3.5 mm, the nanofiber production is less by 10–20% due to a reduced amount of polymer holding on the profiled pin surface and the least duration of spinning. With >3.5 mm pin diameter, multiple Taylor cone formation was noticed, and hence, the electrostatic repulsion disturbed the sphere shape Taylor cone formation resulting in uneven fiber morphology^62^. The polymer loading cycle time of the profiled multi-pin spinneret was found to influence the fiber diameter and productivity of the machine. The trial was conducted with 10 wt % PVA and the polymer loading cycle time was in the range of 5–30 sec. When the polymer loading cycle time was increased to 30 seconds, the productivity of the fiber was reduced (0.820 g/h), as shown in Fig. 4a. Due to the long polymer loading cycle time with high voltage, the solution quantity gets exhausted on the profiled pin surface and hence, the fiber production gradually reduces. At the same time, if the duration is reduced to 10 seconds, the production is reduced (0.520 g/h) due to less working time. However, the average fiber diameter was found to marginally increase from 160 to 172 nm for PVA with 30 kV connected voltage. This direct result of availability of higher quantity of polymer for spinning signifies that a bigger initial spinning radius for a few seconds would promote a larger final jet radius and result in bigger fiber diameter, as shown in Fig. 4b. Once the polymer solution gets exhausted, it reduces the jet radius and fiber diameter. Hence the polymer loading cycle time defines the proportion of finer and coarser fiber. Experimental results have shown that higher feed does promote a significant increment in fiber diameter^63^. Further decreasing the duration to 5 seconds, it decreases the life span of Taylor cone resulting in very little fiber production. Hence, it was fixed at 15 seconds for optimum fiber production and for obtaining a finer diameter of the fiber. Based on the polymer and viscosity, the polymer loading cycle time can be varied to achieve smooth and finer fibers.

The profiled pin distance was also studied to investigate the influence of distance on the nanofiber formation. When the distance between pin was increased above 15 mm, the evenness of the mat gradually reduces and the individual pin outputs were isolated (as shown in supplementary SI Fig. 1). At the same time, when it was reduced to below 10 mm, the fiber movement was distracted strongly by electric field^64,65^. Hence it was fixed at 15 mm to analyse the nanoparticle encapsulation effectiveness without any influence from external factors. Also, it was observed that the needle spacing influences significantly in case of the high viscosity polymer spinning compared to medium or low concentration and viscosity polymer. Compared to top-down spinning, the influence of needle spacing on the fiber formation and morphology due to electrical field repulsion is less in the case of bottom-up spinning. Because the produced fiber is always under the action of two balanced forces during its course of travel from Taylor cone to the collector in the top-down electrospinning: the gravitational and electrostatic force. Hence its influence on the fiber interactions in terms of fiber to fiber repulsion, once it is ejected from the Taylor cone, results in uncontrolled fiber movement. In case of PMES, the amount of polymer fluid withdrawn against the gravitational force from the profiled pin surface and the electrostatic force guides it up to collector, hence the formation of beads and droplets due to excess feeding is also reduced which is one of the issues in the conventional top-down electrospinning. The results are in agreement with the findings of Faissal *et al*.^66^.

**Figure 5:**
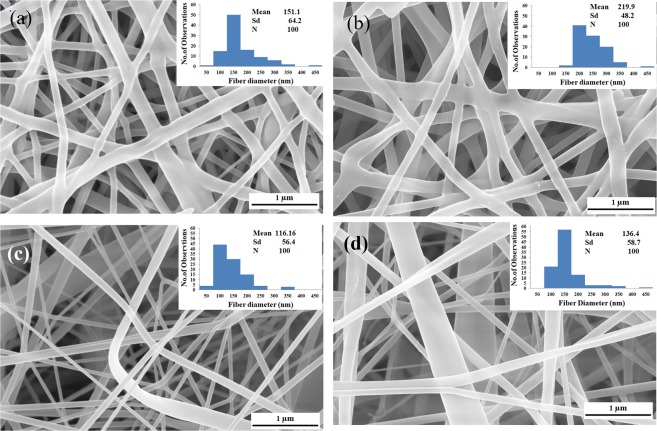
FESEM image of nanofiber produced with the PMES setup with different applied voltages. The images are from PVA and CA polymers and solvents ((**a**) PVA 10 wt % with 35 kV connected voltage (**b**) PVA 10 wt % with 40 kV connected voltage, (**c**) CA 15 wt % with 35 kV connected voltage, (**d**) CA 15 wt % with 40 kV connected voltage. (For 30 kV connected voltage, refer Fig. 7a,c, PVA and CA, respectively).

The connected voltage is also one of the key elements in electrospinning^57,67^. A high applied voltage (>40 kV) is normally required to initiate the upward needleless electrospinning fiber production. The critical voltage requirement is fundamentally related to spinneret design the solution concentration and collector distance^68^. It was found with PMES that when the connected voltage was increased to 25 kV, the spinnerets initiate fiber production, but when it reaches to 30 kV, it achieves the maximum fiber production efficiency at a 12 cm collector distance. By keeping every other parameter at the same level, increasing the connected voltage further to 35 kV leads to decrease in average fiber diameter, but the standard deviation was increased for PVA, since it has an intermittent feeding arrangement. This may be due to the influence of polymer loading cycle and other parameters. Similar trend was observed in both PVA and CA polymer, fiber morphology analysis as shown in Fig. 5a,c. XinWang investigation of the fiber diameter with free surface electrospinning, has shown the slight finer fibers from higher applied voltage^46^; Beyond an optimum voltage (at 40 kV), the fiber diameter increases (Fig. 5b,d) due to the decrease in flight time and probably high voltage is able to draw bigger droplets from the profile pin surface; hence the fiber diameter could be higher^69^.Changing the distance between profiled multi-pin spinneret and collector typically affects the electrical field strength between them, and it requires more applied voltage for fiber production. For PVA, when the distance was increased to 15 cm, a 35 kV connected voltage was required to produce similar fiber production whereas it required 40–45 kV to accomplish the similar fiber development for a 18 cm collector distance. However, beyond a certain range, the fiber formation stopped due to the weakened field strength^70,71^. Similarly, when the distance was reduced to below 12 cm, wet fiber formation was observed. Hence the distance was fixed in the range of 12–15 cm for the optimum applied voltage and the higher productivity. Profiled multi-pin electrospinning also realizes a higher level of productivity than that is feasible with the existing needle electrospinning.

**Figure 6:**
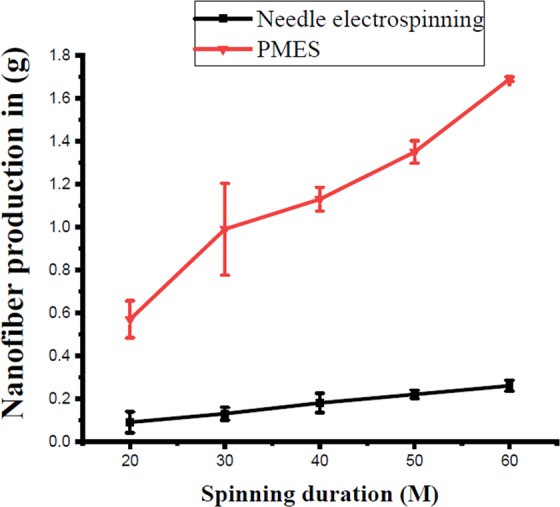
Nanofiber production comparison between PMES and existing single needle electrospinning machines (PVA 10 wt%).

A 10 wt % PVA nanofiber mat was effectively prepared and its productivity without nanoparticle was approximately 1.690 g/h. The produced nanofiber mat as shown in Fig. 2(c), which is approximately 6 or 7 times higher than 0.26 g/h of conventional needle electrospinning productivity^51,72^ with the same PVA polymer concentration and duration, as shown in Fig. 6. Similar production and fiber morphology was achieved while using a stainless steel (SS) multi-pin electrospinning with 35 kV connected voltage. This is due to marginally low conductivity of SS compared to brass^73^. with 10 wt % PVA with 10 wt % zinc oxide nanoparticle, the average production was achieved about 1.780 g/h which is 5–6% higher compared to nanofiber produced without particles due to particle add-on into the fiber. The production values were estimated based on an average of three replications, and it is independent of the particle size and the loading percentage of the particles.

### Approach for encapsulation of nanoparticles

**Figure 7:**
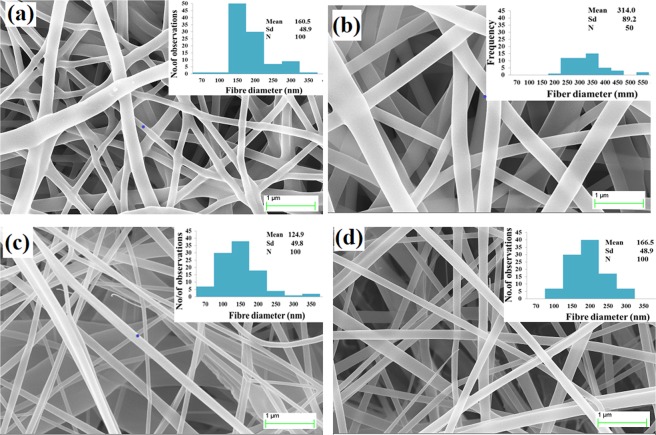
FESEM image of with and without nanoparticle encapsulated nanofiber produced by PMES process ((**a**) PVA without particle electrospun mat (**b**) PVA with Zinc oxide particle electrospun mat (**c**) CA without particle electrospun mat (**d**) CA with Zinc oxide particle electrospun mat.

Figure 7 depicts the FESEM images of the CA and PVA fiber mat with and without Zinc oxide nanoparticle encapsulation produced from the PMES setup. The average fiber diameter was observed as 160.5 ± 48.9 nm for PVA and 124.9 ± 49.8 nm for CA without nanoparticle encapsulation as shown in Fig. 7(a,c) and the diameter can be controlled by changing the spinneret profile and the pin diameter. Figure 7(b,d) shows FESEM image of PVA and CA nanofibers containing 10 wt% with 124-nm size zinc oxide (ZnO) particles and the average fiber diameter was observed to be in the range of 314.0 ± 89.2 nm and 166.5 ± 48.9 nm for PVA and CA, respectively. The add-on particles in the polymer solution were embedded in the produced fiber resulting in increase of the fiber diameter. A linear relation was noticed between the particle size and fiber diameter produced. Also, owing to up and down movement of the spinneret, a continuous turbulence was generated inside the polymer reservoir that resulted in minimising the problem of settling of the particle.

**Figure 8:**
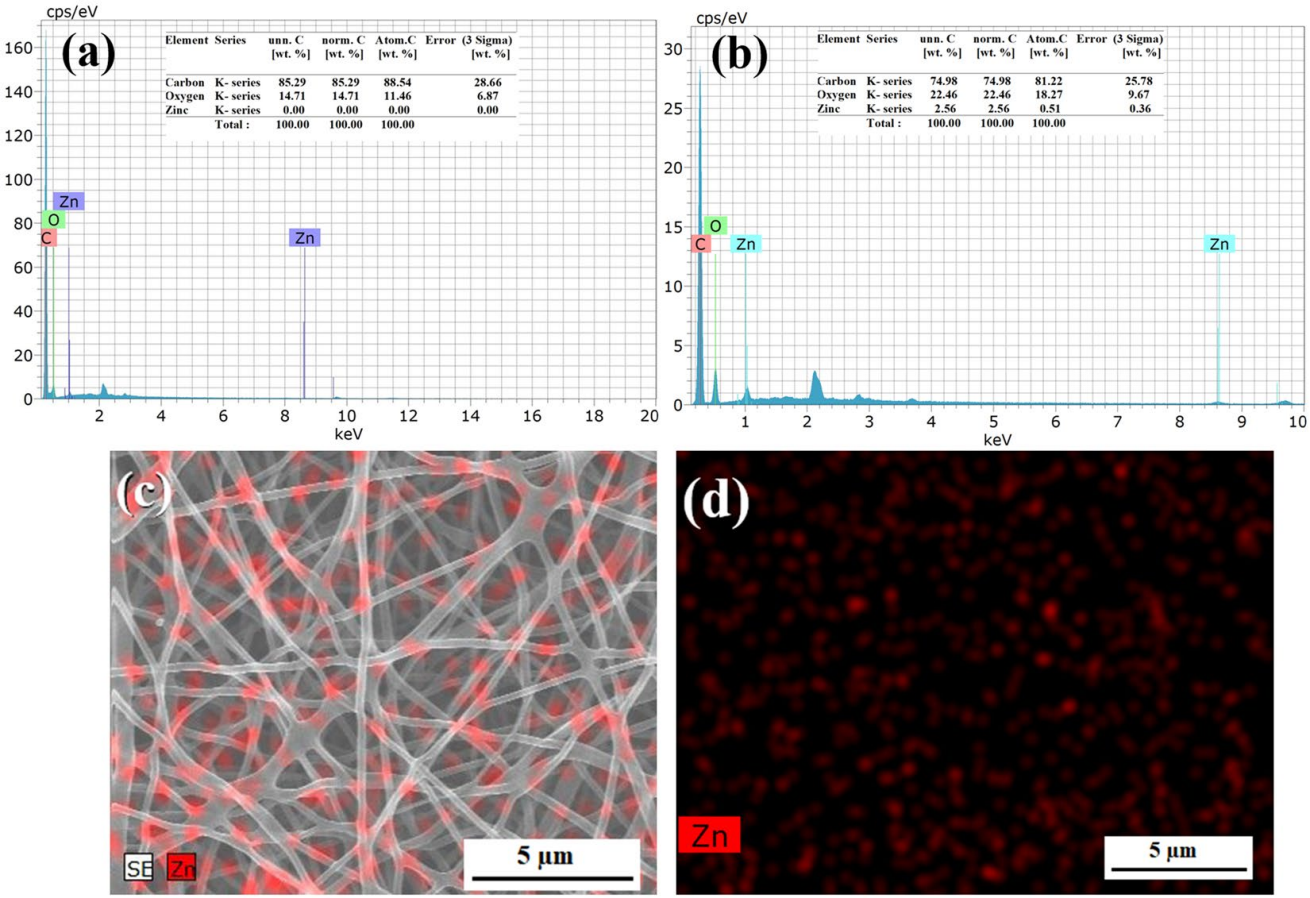
(**a,b**) FESEM- EDX analysis of PVA control and 10 wt % zinc oxide encapsulated PVA electrospun mat (**c,d**) FESEM – EDX elemental mapping of zinc oxide nanoparticle encapsulated with PVA 10 wt % electrospun nanofiber, produced from profiled multi-pin electrospinning setup (PMES).

**Figure 9:**
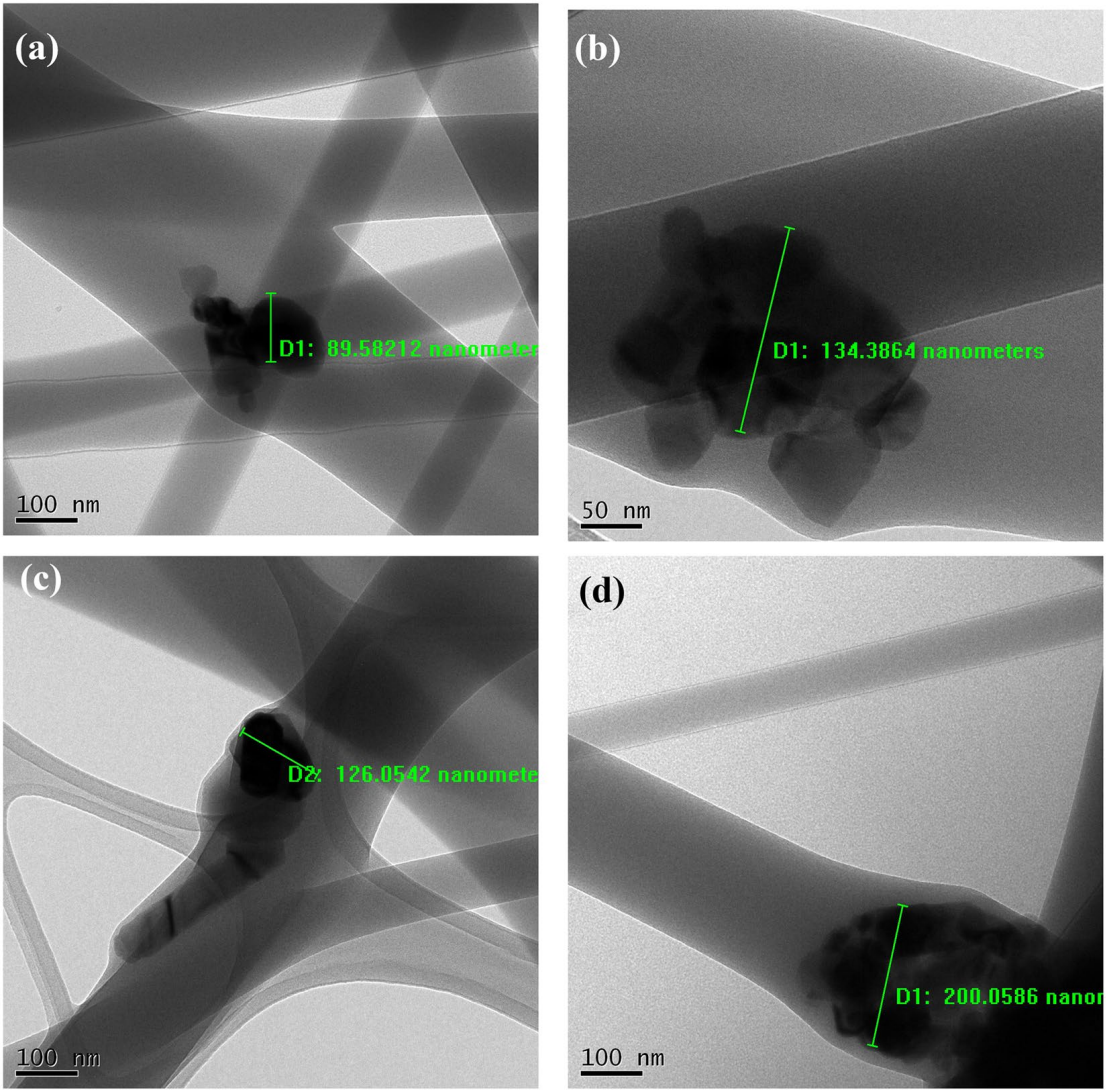
TEM images of ZnO nanoparticles encapsulated with 10 wt% PVA electrospun mat at different places (images a-d).

The EDX spectra for zinc oxide nanoparticle encapsulated PVA electrospun mat and its negative control without zinc oxide electrospun mat is shown in Fig. 8(a),(b). The FESEM- EDX analysis clearly confirms the presence of zinc oxide nanoparticle in the PVA electrospun mat (Fig. 8b) as against a sample (without nanoparticle) (Fig. 8a). Also, it was confirmed through elemental mapping, that the encapsulated nanoparticles are equally dispersed into the PVA nanofiber mat throughout the fiber production process, without any aggregation (Fig. 8c,d). Further, ZnO nanoparticle loading and distribution on the PVA nanofiber mat were observed by Transmission Electron Microscope (TEM) (Fig. 9). The average size of the ZnO particle within the PVA electrospun fiber was observed as 116 ± 19.54 nm and it is in concurrence with the XRD investigation. It shows that the ZnO nanoparticles were encapsulated along with the PVA polymer at the time of electrospinning.

## Conclusion

We have demonstrated a prototype version of the novel design to produce nanoparticle encapsulated electrospun mat using profiled multi-pin spinnerets. This technique combines the advantages of both the needle and needleless electrospinning systems, where the sphere-shaped polymer profile created to facilitate Taylor cone formation similar to needle electrospinning with an increased productivity comparable to the needleless electrospinning system. The new design can handle high viscosity polymers and produce nanoparticles encapsulated nanofibers. Primarily, the system productivity is restricted by the number of profiled pins present in the spinneret. In prototype design, 21number of pins were used and achieved the production of 1.780 g/h with solution concentration of 10 wt % PVA with 10 wt% zinc oxide nanoparticles, applied voltage of 30 kV, collector distance of 12 cm. This work gives a framework for designing a high production electrospinning machine for the production of functional composite nanofibers with embedded nanoparticles. Future research will focus on the scaling up the pins, continuous polymer feeding on the profiled pin surface and a different size of polymer droplet influencing the morphology of the resulting fibers and productivity to enable the successful manufacturing and commercialization.

## Methods

### Profiled multi-pin electrospinning apparatus

The apparatus consists of 21 number of profiled multi-pins of 25 mm length and 3.5 mm diameter, made of brass metal were fixed in a circular disc at a distance of 15 mm from each other. Collectively, these are referred to as the profiled multi-pin spinneret assembly. The assembly was connected with the pneumatic cylinder for providing up and down motion to the spinneret for continuous refilling of the polymer solution from the bottom of the reservoir. During up and down motion of the spinneret assembly, the spinneret was immersed in the polymer solution and due to the profiled pin surface and surface tension of the polymer; a sphere shaped polymer droplet was created on the pin surfaces. When the sphere-shaped droplet solution was exposed to a high voltage, Taylor cones were formed on the profiled pin surface and fibers were collected on the bottom of the collector. The formation of this eruption focuses was guided by the electric field. In the arrangement, the spinneret to collector distance can be adjusted from 50–250 mm by a threaded rod and the spinneret up and down movement cycle timing can be varied from 0–180 sec through an electrical circuit.

### Polymer solution preparations

Fully hydrolyzed polyvinyl alcohol (PVA) polymer (Mw = 70,000–100000 g/mol obtained from Himedia®, India) was dissolved in deionized water at 90 ° C for 2 hours to attain the maximum viscosity when it becomes completely homogenous to produce 8, 10, 12 wt% solutions. Cellulose Acetate (CA) polymer (Mw = 30000, Sigma Aldrich) was dissolved in Dimethylformamide (DMF) and acetone in the ratio of 1:2. to produce 12, 15, 18 wt% solutions.

### Nanoparticle synthesis

The nanoparticle was synthesized with the help of soluble starch as a stabilizer, the procedure followed was similar as reported earlier^74^. Based on the XRD diffraction analysis, the average particle size was measured as 124 ± 10 nm (XRD Peaks as shown in supplementary SI Fig. 2).

### Nanofiber preparation using polymer solutions

The nanofibers were produced from the profiled multi-pin electrospinning machine. The newly designed machine consisted of a DC voltage source, polymer reservoir, profiled multi-pin spinneret assembly, where the sphere-shaped polymer jets and Taylor cones were formed and fibers were collected in a grounded flat metal collector (Fig. 1a,b). The polymer solution loaded on the reservoir was, subsequently, loaded on the profiled pin spinneret surfaces by the up and down motion of the spinneret assembly. The up and down motion was controlled through electrical timer circuit and the timing was fixed as 15 sec and 2 sec for upward and downward movement, respectively. The charged polymer solution was attracted by the collector and fibers are formed during travel between spinneret to the collector. The supplied voltage and distance between the collector and the profiled multi-pin spinneret surface were set as 30 kV and 12 cm, respectively. For every cycle, the polymer loading cycle time of the multi-pin profiled spinneret assembly was kept at 15 sec.

### Characterization of nanofibers

The prepared nanofiber surface morphology was analyzed using Transmission Electron Microscopy (TEM), Field Emission Scanning Electron Microscopy (FESEM) and energy dispersive X-ray analysis (EDX) system. The EDX mapping was coupled with FESEM for particle analysis and the diameters were calculated by using the ImageJ® software. The samples were coated with gold to prevent them from getting charged and forming a source of reflecting electrons that may prompt to recording of poor quality of the image in FESEM.

## Supplementary information

Supplementary information.

Video 1 
