# Supplementary material for: A Novel Profiled Multi-Pin Electrospinning System for Nanofiber Production and Encapsulation of Nanoparticles into Nanofibers
Source: Sci Rep. 2020 Mar 9;10:4302. doi: 10.1038/s41598-020-60752-6 (PMC7062762; doi:10.1038/s41598-020-60752-6)
Supplement: Supplementary file 2 — Video 1 [file 41598_2020_60752_MOESM2_ESM.doc]

***A Novel Profiled Multi-Pin Electrospinning System for Nanofiber Production and Encapsulation of Nanoparticles into Nanofibers***

**G.T.V. Prabu*^1^, Bhaarathi Dhurai^2^**

^*1^ICAR-Central Institute for Research on cotton Technology, Mumbai, India. ^2^Department of Fashion Technology, Kumaraguru College of Technology, Coimbatore, India. These authors contributed equally to this work. Correspondence and requests for materials should be addressed to G.T.V. Prabu. (email:geeteevee@gmail.com)


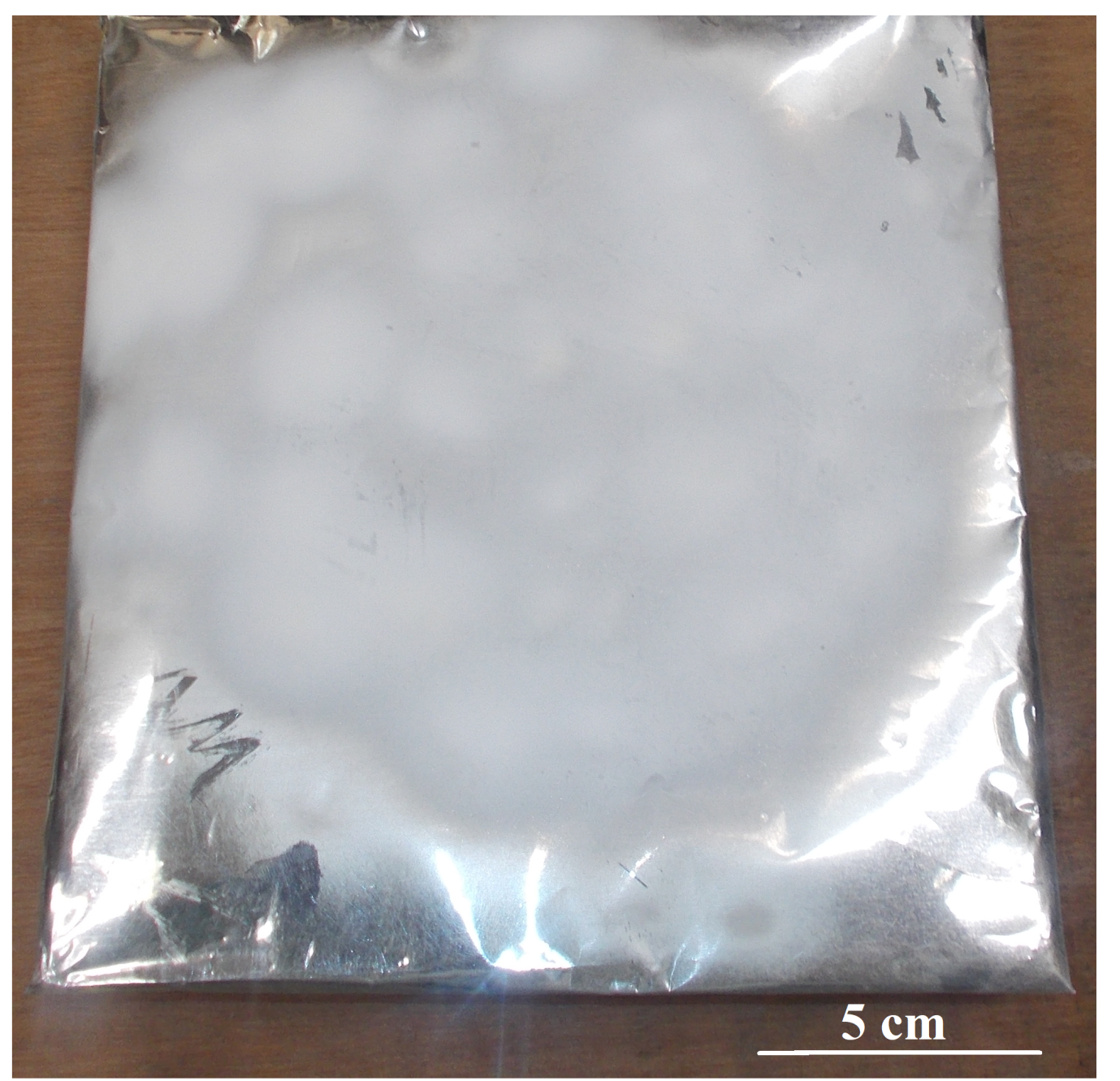


SI Figure :1 Image of 10 wt % PVA electrospun mat produced from PMES with above 15 mm profiled pin distance.


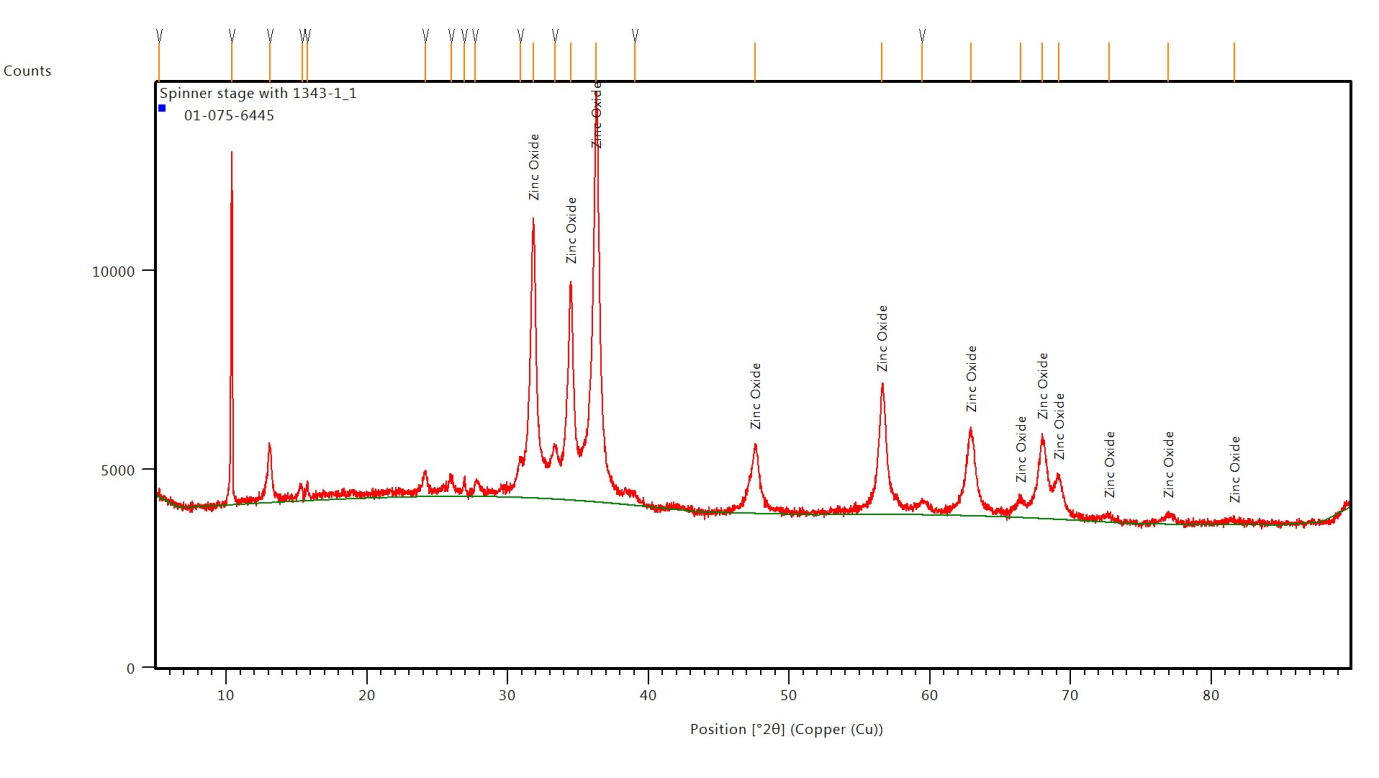


SI Figure 2: XRD pattern of ZnO nanoparticles with 0.5 % starch, the peaks assigned to diffractions from various planes are of hcp Zinc oxide.
